# Seasonal variations of microbial community structure, assembly processes, and influencing factors in karst river

**DOI:** 10.3389/fmicb.2023.1133938

**Published:** 2023-03-23

**Authors:** Xiangyu Guan, Ruoxue He, Biao Zhang, Chengjie Gao, Fei Liu

**Affiliations:** ^1^School of Ocean Sciences, China University of Geosciences, Beijing, China; ^2^Department of Discipline Construction and Technology Development, Chengdu Technological University, Chengdu, China; ^3^Beijing Municipal Research Institute of Eco-Environmental Protection, Beijing, China; ^4^Key Laboratory of Groundwater Conservation of MWR, China University of Geosciences, Beijing, China

**Keywords:** karst river, microbial community, assembly, nitrogen, antibiotic

## Abstract

The physicochemical properties and microbial communities have significant annual and seasonal changes in karst aquifers. To explore the changes of microbial community and their relationships with environmental factors, water samples were collected from a typical karst river. Microbial communities in winter (Jan-2017 and Jan-2019) were stable with high similarity in spite of the 2 years sampling interval, but the microbial communities in Aug-2017 was different from that in Aug-2018. In four sampling times, there were 275 shared genera, whose average relative abundance ranging from 89.04 to 96.27%. The winter and summer specific genera were mainly from the recharge of tributary site K6 and discharge of waste water treatment plant (K2 and K3), respectively. The deterministic processes had a more significant effect on the microbial community assembly in winter than that in summer, which was affected by environmental pressure from pollution. Furthermore, antibiotics and inorganic nitrogen pollution affected element cycles of nitrogen and sulfur indirectly through microbial ecological modules in karst river, and the denitrification and desulfurization processes were potentially inhibited. These findings contributed to understand the changes and its assembly mechanism of microbial community, as well as the feedback to environment in polluted karst river.

## 1. Introduction

Approximately 15% of the continents are karst terrain (Yuan, 1997). The water cycle was rapid in the karst system because of its underground-surface double-layered structure and high hydraulic conductivity (Torres et al., 2018). Due to the uneven distribution of karst aquifer fissures and tubes, rapid discharge, and significant seasonal flow velocity variation, the hydrological processes and hydrochemical characteristics exhibit substantial spatial and temporal variability (He et al., 2018; Sun et al., 2019, 2021). Karst water provided drinking water for about 25% of the global population (Dodgen et al., 2017). With the strengthening human activities, however, pollutants such as nitrogen, phosphorus, heavy metals, and organic matter (Wu et al., 2018; Huang F. et al., 2019; Qin et al., 2020, 2021) are continuously introduced into karst aquifers, which have altered the karst ecosystem and element cycles.

As the most active component of the aquatic ecosystem, microorganisms acquire nutrients and energy to survive in an oligotrophic environment *via* numerous metabolic pathways, which are crucial to the elemental cycling of the karst ecosystem (Webster et al., 2018; Xue et al., 2020; Zhu et al., 2022). According to several studies, Proteobacterium, Bacteroides, and Firmicutes were the most common phyla in karst aquifers (Li et al., 2017; Danczak et al., 2018; Tang et al., 2019; Li Q. et al., 2020), and there were shifts in both geographical and seasonal composition of microbial communities (Opalički Slabe et al., 2021). Deterministic and stochastic processes jointly govern the assembly of microbial communities (Chave, 2004). Previous studies have demonstrated that the relationship between these processes varies across different spatial and temporal scales due to the intensity of environmental change and the threshold of microbial tolerance (Liu et al., 2019). In karst aquifer, the relatively steady hydrodynamic circumstances during the dry season resulted in the distinct microbial communities in individual aquifers because of their distinct physicochemical conditions (Shabarova, 2013; Shabarova et al., 2014), whereas the rapid recharge during the rainy season enabled the passive migration of isolated species in densely connected aquifers, reducing the environmental selection on microbial communities and resulting in their convergence in karst aquifer (Yan et al., 2020). In addition to the aquifer connectivity, different hydrochemical conditions affected the composition and distribution of “permanent inhabitants” and specific species in different seasons in microbial communities. In the unexplored karst aquifer, pH, temperature (Yun et al., 2016; Li H. et al., 2021), salinity (Park et al., 2021; Fang et al., 2022), and organic matter (Iker et al., 2010) were the most impacted factors on the microbial communities. With increasing human activities, the constant input of nitrogen, phosphorus and emerging pollutants such as antibiotics had disrupted the physicochemical properties, the structure and interactions of microbial communities, as well as their carbon and nitrogen cycles (Zhang et al., 2013; Xi et al., 2015; Mahana et al., 2016; Zhernakova et al., 2016; Subirats et al., 2019; Roose-Amsaleg et al., 2021). Not only had antibiotics affected the microbial community structure, but also influenced their metabolic mechanisms, such as carbon sequestration, nitrification and denitrification (Gonzalez-Martinez et al., 2014; Xiong et al., 2015; Li H. et al., 2020; Slipko et al., 2021). Different bacteria acquired resistance genes variously under the selection pressure caused by antibiotics, which regulated their metabolic strategies and the efficiency of element cycles (Ming et al., 2020; Yang et al., 2020; Zhang et al., 2022). However, karst aquifers with intense human activities were often influenced by more than one pollutant (Zhu et al., 2019), which would have more complex effects on the community structure and function of microbial community.

The elements cycles were driven by biogeochemical processes in karst system, which would further influence the role of karst system in global climate change (Mahler et al., 2021). However, the microbial assembly and ecological roles in polluted karst rivers at annual and seasonal scales, as well as their feedback to environment were less studied. In this study, we focused on: (1) the physicochemical properties and pollution characteristics of typical karst river in dry and rainy seasons; (2) the variation and assembly mechanisms of microbial communities in polluted karst river; and (3) the response of microbial community composition and functions to environmental factors, especially the pollutants from human activities.

## 2. Materials and methods

### 2.1. Study area and sample collection

**Figure 1:**
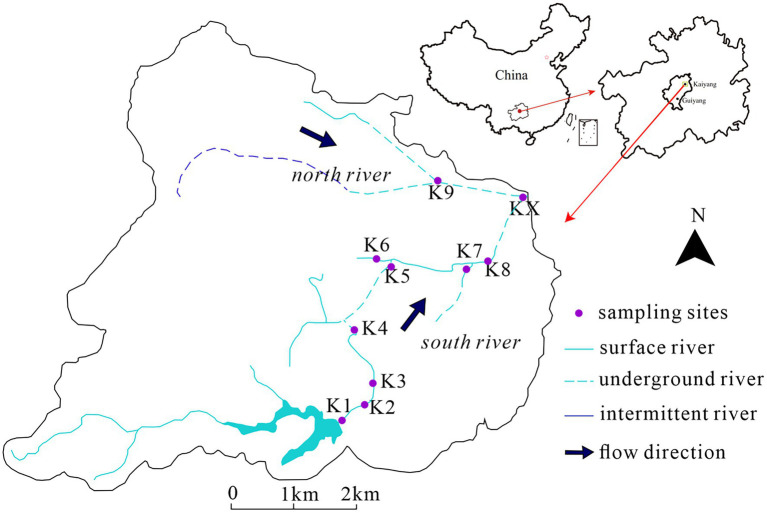
The sampling sites in study area.

The study area, Kaiyang, is situated in Guizhou Province, southwest China, in a typical subtropical monsoon climate zone with an annual average temperature of 14.4°C and precipitation primarily concentrated from June to September. There were two karst rivers from west to east, the south river and the north river, with three underground entrances (K4, K8, K9) and three underground exits (K5, K7, and KX) (Figure 1). A total 40 samples were collected. Each 10 samples were collected in January 2017 (Jan-2017), August 2017 (Aug-2017) (Xiang et al., 2020), August 2018 (Aug-2018) and January 2019 (Jan-2019) (Zhang et al., 2021), respectively.

Except for K9 and KX, all sampling sites were in the south river. K1 to K4 were located in the surface river from upstream; K1 was located in a reservoir; K2 and K3 were situated upstream and downstream of a waste water treatment plant (WWTP), respectively; and K4 was situated in a resort. There were agricultural fields and a small amount of livestock activity between K5 and K8. K5 was an outlet of underground river, with evident domestic wastes. K8 was an underground entrance. K6 and K7 were located in the upstream tributaries of K5 and K8, respectively. K6 was a surface river site with domestic wastes, and K7 was the outlet of an underground river. K9 was a sinkhole in the scenic region of the north river, while KX was the confluence of the north and south rivers.

### 2.2. Hydrochemistry monitoring and analyses

Temperature (T) and pH value of karst river water samples were determined by pH meter (pH30, CLEAN, CA, United States). Electronic conductivity (EC) and dissolved oxygen (DO) were determined by EC meter (CON, CLEAN, CA, United States) and DO meter (CON30, CLEAN, CA, United States), respectively. The total organic carbon (TOC) was determined by TOC analyzer (Shimadzu, Japan). HCO_3_^−^ was tested by potentiometric titrator (Metrohm 877 Titrino plus, Swiss), and Cl^−^, SO_4_^2−^ were measured by ion chromatograph (DIONEX ICS-900, Sunnyvale, CA, United States). Ca^2+^, Mg^2+^, K^+^, and Na^+^ were measured by ICP-OES (SPECTRO Blue Sop, Germany). And the spectrophotometer UV-1800 (Shimadzu, Japan) was used to determine dissolved inorganic nitrogen (DIN) - NH_4_^+^, NO_3_^−^ and NO_2_^−^. Following our previous studies (Huang H. et al., 2019), auto-solid phase extraction (SPE) instrument (Auto SPE-06C, Reeko Instrument, TX, United States) with an Oasis HLB SPE column (6 mL, 500 mg, Waters, MA, United States) was used to extract antibiotics from water samples of karst river. Samples were collected and immediately chilled in 4°C and transported *via* overnight express to the laboratory for further test. The sampling process was followed our previous study (Xiang et al., 2020).

### 2.3. DNA extraction and 16S rRNA gene sequencing

The bacteria in each sample were filtered through a 0.22 μm Millipore GSWP membrane for 1.0 l water and then suspended in 10 mL of physiological saline for 6 h. The mentioned suspensions were separated and kept for DNA extraction using a PowerSoil DNA Extraction Kit (MoBio Laboratories, Carlsbad, CA, United States). The extracted DNA was stored at −20°C for further analysis and at −80°C for permanent preservation. Using a NanoDrop ND-2000 spectrophotometer (Thermo Fisher Scientific, Wilmington, DE, United States) and agarose gel electrophoresis (Bio-Rad, Hercules, CA, United States), the quantity and quality of the extracted DNA were established.

The primers 338F and 806R (338F: 5′-barcode-ACTCCTAC GGGAGGCAGCAG-3′ and 806R: 5′-barcode-GGACTACHVGG GTWTCTAAT-3′) were used to amplify the V3-V4 regions of the bacterial 16S ribosomal RNA gene (16S rRNA gene). The PCR amplification method was referred to our previous study (Zhang et al., 2020). Raw fastq files were demultiplexed, then quality-filtered using QIIME (Version 1.17). All clusters were subsampled by the minimum reads (19,551 reads), and operational taxonomic units (OTUs) were clustered with a 97% similarity cutoff by Uparse.^1^ Mothur^2^ was used to compute the alpha diversity. The Kyoto Encyclopedia of Genes and Genomes (KEGG) functional profiling was predicted using PICRUSt2.^3^ The clustered sequence datasets in this study were deposited in the NCBI Genbank with the accession number PRJNA908735 and PRJNA936825.

### 2.4. Statistical analysis

The data were normalized except for pH value. Spearman correlation analysis and its significance test among each factor were completed by “corrplot” package.^4^ The ordinary least squares (OLS) analysis among ammonium, nitrate, sulfate, and other environmental factors were calculated by “car” package.^5^ Principal co-ordinates analysis (PCoA) based on Bray-Curtis distance and variance partitioning analysis (VPA) were both performed by “vegan” package.^6^ Venn diagram at genus level was plotted by “vennDiagram” package. The Kruskal-Wallis test (*p* < 0.05) was completed by “nparcomp” package, and corrected *p*-values were calculated using Dunn’s test. The microbial community assembly processes were analyzed by “iCAMP” package.^7^ And the heatmap figure was plotted by “pheatmap” package.^8^ All the above data analysis were implemented in R.^9^ The co-occurrence network of bacteria at genus level was visualized with Gephi 0.9.2,^10^ and ecological modules were identified using the Louvain algorithm. The saturation index of gypsum (SIg) and saturation index of calcite (*SIc*) were calculated by PHREEQC (Version 3.6.2). Structural equation model (SEM) was performed by SPSS AMOS (Version 24.0) to discuss the environmental factors effect on ecological module and element metabolic module.

## 3. Results and discussion

### 3.1. Characteristics of physicochemical properties of karst river

The pH value of karst river water was ranging from 7.70 to 8.82, and the temperature was ranging from 4.0°C to 26.1°C (Supplementary Table 1). DO ranged from 1.88 to 9.93 mg/L, and TOC ranged from 1.09 to 30.63 mg/L. Ca^2+^ and HCO_3_^−^ were the major cation and anion due to the abundant carbonate rocks in the study area (Sun et al., 2019). For carbonate rock weathering involving only carbonic acid, the equivalent ratio of [Ca^2+^+Mg^2+^]/[HCO_3_^−^] is typically around 1. But the average value of our study was 1.23, while the mean value of [Ca^2+^+Mg^2+^]/[HCO_3_^−^ + NO_3_^−^ + SO_4_^2−^] was 0.93, indicating that the carbonate weathering was influenced by both carbonic acid and sulfuric/nitric acids (Martin, 2017; Sun et al., 2021).

The concentration of NO_3_^−^ varied from 0.74 to 45.82 mg/L, with an average value of 18.23 mg/L, and the concentration of SO_4_^2−^ varied from 20.14 to 74.25 mg/L. There were severe NH_4_^+^ and NO_2_^−^ pollution, with NH_4_^+^ amounting from 0.02 to 45.35 mg/L and NO_2_^−^ amounting from 0.01 to 9.51 mg/L. The concentrations of both NH_4_^+^ and NO_3_^−^ showed seasonal variations with higher in winter and lower in summer, mainly influenced by the dilution effect of precipitation recharge in rainy season. Meanwhile, the NH_4_^+^ were higher in upstream, particularly from K1 to K4, while NO_3_^−^ concentrated downstream from K7 to KX, due to nitrification and the NO_3_^−^ accumulation for its high solubility (Li X. et al., 2021; Li H. et al., 2021). The negative correlations between NH_4_^+^ and NO_3_^−^ also supported this hypothesis (Eqs 1, 2):


(Equation 1) $$\begin{array}{l} {\text{NH}}_{4}^{+}=-0.35{\text{NO}}_{3}^{-}+0.17\text{TOC}-0.31T\\ \;+0.20\text{DO}+0.50\text{Antibiotics}\\ \;+a1(R2=0.56,p<0.01,a1<0.0001) \end{array}$$

(Equation 2) $$\begin{array}{l} {\text{NO}}_{3}^{-}=-0.42{\text{NH}}_{4}^{+}+0.54{\text{NO}}_{2}^{-}-0.60T+0.27\text{pH}\\ \;+a2\;(R^{2}=0.48,p<0.01,a^{2}<0.0001). \end{array}$$

As shown in Eq. 1, The concentration of NH_4_^+^ was positively correlated with antibiotics, as well as its correlation coefficients with macrolides, lincomycin, tetracyclines were 0.78, 0.71 and 0.66 (*p* < 0.01), respectively. The concentrations of NH_4_^+^ and antibiotics showed a similar spatial variation trend, displayed higher upstream. As one of the most prevalent forms of nitrogen pollution in WWTP, NH_4_^+^ originated primarily from wastewater plant discharges, the same source of antibiotics.

SO_4_^2−^ is mainly derived from both natural (dissolution of soluble sulfate, oxidation of sulfide minerals, and atmospheric precipitation) and anthropogenic inputs(agricultural fertilizers, domestic sewage, industrial wastewater, and mine waste water)(Li et al., 2018). In the study area, the [Mg^2+^]/[Ca^2+^] values ranged from 0.39 to 1.14 with average 0.65, which indicated that the chemical composition is mainly influenced by low-magnesium minerals such as calcite and gypsum. Furthermore, the SIg values were − 2.43 ~ −1.75 while *SIc* were − 0.79 ~ 1.28, implied that the dissolution of gypsum were the main source of sulfate. Additional, SO_4_^2−^ were positively correlated with antibiotics (*R* = 0.66, *p*<0.01), which implied that SO_4_^2−^ also originated from WWTP, which was consistent with its concentration spatial distribution of higher in upstream.

**Figure 2:**
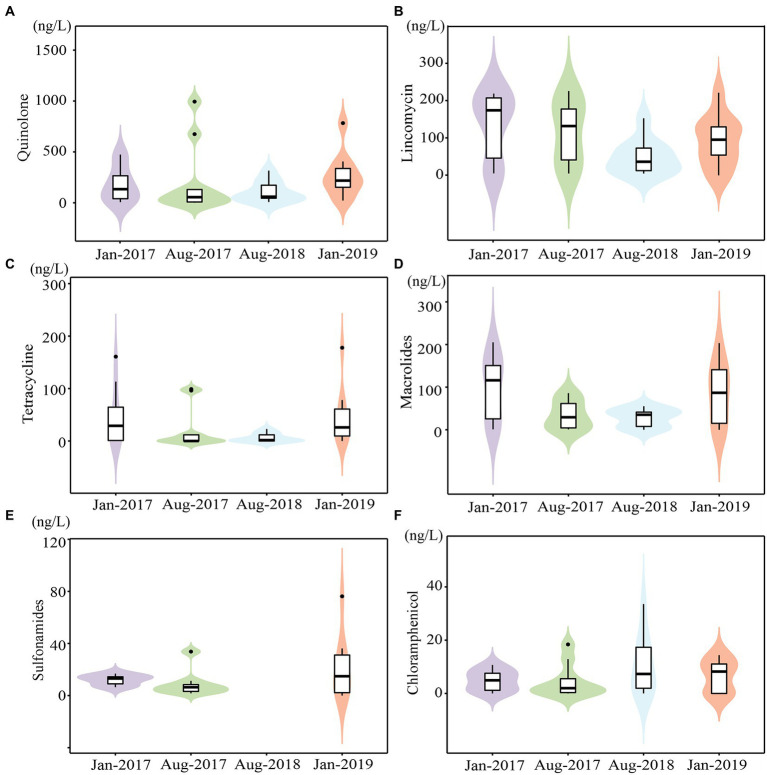
The concentrations of main antibiotics in karst river at four sampling times (Jan-2017, Aug-2017, Aug-2018 and Jan-2019). **(A–F)** The concentrations of quinolones, lincomycin, macrolides, tetracyclines, sulfonamides and Chloramphenicol.

A total of 28 antibiotics were detected, with total concentrations ranging from 9.13 to 1411.33 ng/L (Supplementary Table 2). The mean concentrations of four sampling times from high to low were Jan-2019 (average value of 514.15 ng/L), Jan-2017 (472.60 ng/L), Aug-2017 (392.44 ng/L), and Aug-2018 (199.37 ng/L), with a seasonal trend of higher in winter and lower in summer (Figure 2), due to the dilution effect of precipitation recharge, which was consistent with our previous study (Huang F. et al., 2019; Huang H. et al., 2019). The maximum value for each time was frequently recorded at K2 and K3, suggesting that WWTP effluent discharge was the major source of antibiotics in karst river, which indicated the anthropogenic inputs. Antibiotic demonstrated a spatial changes that fluctuates and decreases along the stream, which was affected by the adsorption of soil and sediment and the degradation of bacteria (Zhao et al., 2012). Moreover, antibiotic concentrations in tributaries K6 and K7 were lower than those in the mainstream.

Quinolones (QNs), lincomycin (LIN), and macrolides (MLs) were the major antibiotics, accounting on average for 43.45, 34.58, and 13.99% of total concentration, respectively, which were consistent with their frequent use in southwest China (Sun et al., 2015; Zeng et al., 2019). QNs ranged from 3.10 to 994.13 ng/L (Figure 2A) with a 95% detection frequency. QNs were persistent pollutants due to their high chemical stability and prolonged half-life (Andriole, 1999). Ofloxacin and norfloxacin, as the maximum content subclass of QNs in most WWTP (Dinh et al., 2017), also dominated in karst river, ranging from 4.60 to 486.00 ng/L and 4.09 to 204.00 ng/L, respectively. The detection frequency of LIN was 95%, and the concentration was 4.77–227.53 ng/L (Figure 2B). The detection frequency of MLs was 88%, with concentration ranging from 0.83 to 205.13 ng/L (Figure 2C). Erythromycin and roxithromycin were the main subclasses of MLs. While antibiotics were transferred underground by continuous precipitation recharge in summer, their natural degradation was weakened due to weak light and then accumulated (Liu et al., 2021). However, the high connectivity of karst aquifers enabled rapid hydration and a more quickly response to contaminants.

The concentrations of tetracyclines (TCs), chloramphenicols (CAP), and sulfonamides (SAs) were ranged from 3.43 to 177.80 ng/L (Figure 2D), 1.70 to 76.20 ng/L (Figure 2E), and 0.65 to 33.56 ng/L (Figure 2F), respectively. The detection rate of TCs was 60%, with tetracycline and oxytetracycline being the most commonly used antibiotics. The detection frequency of CAP was 53%. Both TCs and CAP were higher in winter. The detection rate of SAs was 75%, with sulfapyridine being the most common subclass. The maximum content and the most subclasses of SAs were observed in Aug-2018. Only samples from Aug-2018 contained sulfadimethoxine and sulfadimidin, both of which were widely applied in veterinary medicine. Increased livestock vaccination in study area in 2018 may result in an increase use of veterinary drugs.^11^ As a notable veterinary drug, enrofloxacin was also detected only in Aug-2018.

### 3.2. The structure of bacterial community in karst river

A total of 3,955,799 optimum sequences were obtained in 40 samples (Supplementary Table 3). After subsampling by the minimum sequences, 5,242 OTUs were obtained. The species richness in Aug-2017 was the lowest. Nonetheless, the Shannon index had no significant difference at four sampling times (Supplementary Table 3).

Proteobacteria (relative abundance ranged from 36.03 to 61.15%), Bacteroidetes (18.97 to 38.03%), Cyanobacteria (6.14 to 13.85%), and five other phyla with relative abundance greater than 5% were the dominant phyla. *Flavobacterium*, *Arcobacter*, norank_c_Cyanobacteria, *Pseudomonas* and *Acinetobacter* were prevalent in a total 945 genera, with the relative abundance of 12.68, 5.78, 5.18, 4.41 and 4.35%, respectively.

**Figure 3:**
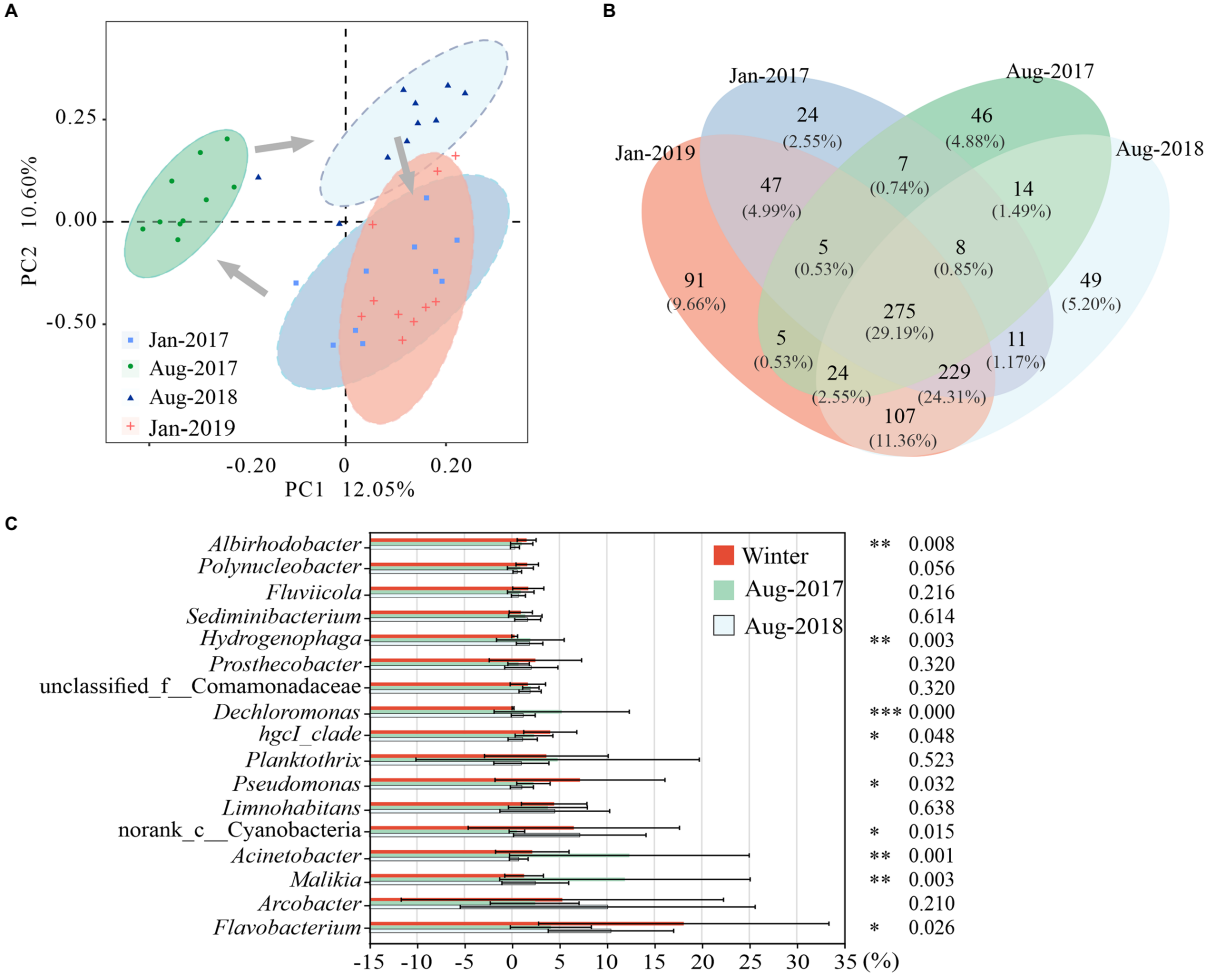
Differences of microbial communities in karst rivers among four sampling times (Jan-2017, Aug-2017, Aug-2018 and Jan-2019). **(A)** Principal co-ordinates analysis (PCoA) of microbial community structure based on Bray-Curtis distance at genus level. **(B)** Venn diagram of microbial community at genus level. **(C)** Significantly Different of genus among two winter, Aug-2017 and Aug-2018. Kruskal-Wallis test with *p*-values were *p < 0.05, **p < 0.01, ***p < 0.001.

PC1 and PC2 explained 22.65% of the variance in community structure together (Figure 3A). The bacterial communities of Jan-2017 and Jan-2019 were similar. The bacterial community structures of Aug-2017 and Aug-2018 were divided from each other, which apparently differed from samples taken in winter.

The relative abundance of 275 shared genera varied from 89.04 to 96.27% (Figure 3B). Most of them showed obvious seasonal characteristics. Seven genera were mainly distributed in winter, including *Flavobacterium*, *Pseudomonas* and *hgcI_clade*. In previous study, we observed strong correlations between ARGs and *Flavobacterium*, *Pseudomonas* (Zhang et al., 2021), which were more likely to survive in winter with higher antibiotic concentrations. Six genera were enriched in summer, including *Dechloromonas*, *Hydrogenophaga* and *Cloacibacterium*. The relative abundance of unclassified_f__Comamonadaceae gradually increased while *Limnohabitans* showed no noticeable trend in this study. Nine out of the nineteen dominant genera (relative abundance above 1%) (Supplementary Figure 1) exhibited statistically significant differences (*p* < 0.05) (Figure 3C). *Flavobacterium*, *Pseudomonas*, *hgcI_clade* and *Albirhodobacter* were considerably enriched in winter with average relative abundances varying from 8.15 to 28.06%, 6.26% to 8.08, 3.53 to 4.54% and 1.49 to 1.62%, respectively. *Acinetobacter*, *Malikia*, *Dechloromonas* and *Hydrogenophaga* were highly expressed in Aug-2017 with average relative abundances of 12.37, 11.90, 5.24 and 1.94%, respectively. And the relative abundance of norank_c_Cyanobacteria (7.15%) was the highest in Aug-2018.

Compared with shared genera, the richness and abundance of specific genera were lower. Forty seven genera only presented in winter, with relative abundances of 0.23 and 0.24% in Jan-2017 and Jan-2019, including Candidatus_Azambacteria, *Empedobacter*, etc. The winter genera were mainly distributed at K4 and K6 in Jan-2017 and at K6 in Jan-2019. As a sampling site in the tributary, there might be a relatively higher flow at K6 that introduce a great number of exotic species in winter. Forty six genera were only found in Aug-2017, with an average relative abundance of 1.13%, and distributed mainly at K2 and K3, such as *Macromonas*, Candidatus_Aquiluna. Forty nine genera such as *Sporacetigenium* and *Desulfitobacterium* were only found in Aug-2018, with an average relative abundance of 1.73% and primarily enriched at K8.

**Figure 4:**
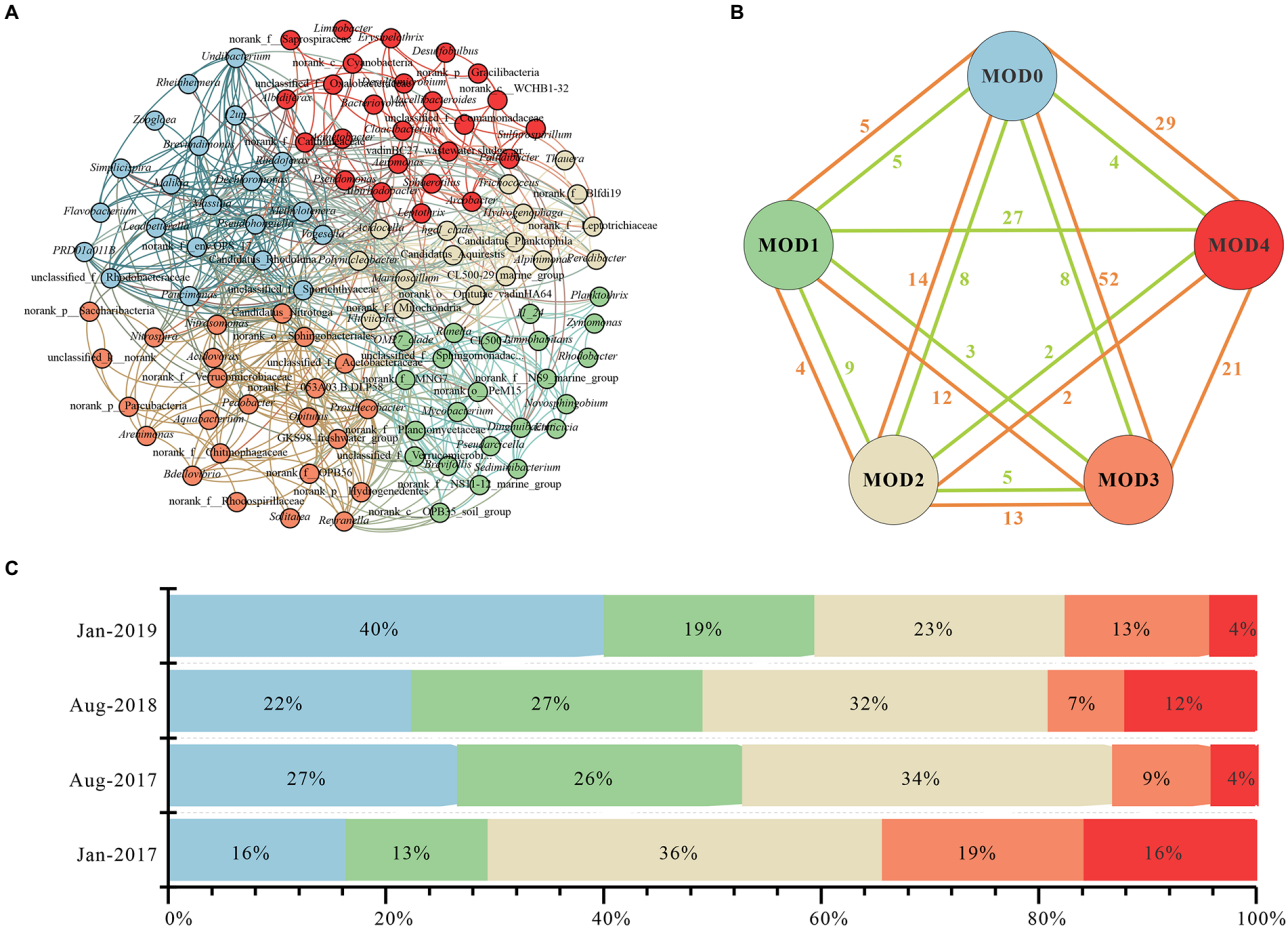
Interactions of microbial community in karst river. **(A)** Co-occurrence network of genus with relative abundance above 0.1%. Genus with relative abundance above 0.1% were selected to calculate their correlations. Each node represented a genus, the nodes were colored by module, and the connection between nodes represented a significant correlation (Spearman, *p* < 0.05, |*r*| > 0.6). **(B)** Connections of modules. The correlation of each genus in every module, negative relations were represented in green edges and positive relations were represented in orange edges. **(C)** Stacked histogram of five modules in Jan-2017, Aug-2017, Aug-2018, and Jan-2019. Relative abundance of each module was represented in different color.

Five clusters (MOD0-MOD4) were obtained through co-occurrence network analysis of genera with relative abundance above 0.1% (Figure 4A). Twenty one genera were included in MOD0, primarily *Flavobacterium*, *Malikia*, *Dechloromonas*, etc., each of which exhibited distinct seasonal differences. Thus, there was no discernible seasonal trend in MOD0 but a peak value in Jan-2019 (Figure 4C), where *Flavobacterium* concentrated. Twenty three genera of MOD1 were mainly included *Limnohabitans* and *Planktothrix*, which concentrated in summer. Twenty five genera were included in MOD2, such as *Arcobactor*, *Pseudomonas*, *Cyanobacteria*. MOD3 was consisted of 17 genera, such as *hgcI_clade* and *Hydrogenophaga*, etc., which were abundant in winter. *Prosthecobacter*, *Nitromonas* and *Nitrospira* were dominant in MOD4 among the total 24 genera.

The co-occurrence network of major microorganisms helped to reveal functional group structure and potential interactions between bacteria (Curtis et al., 2002; Cardona et al., 2016). There were 165 positive and 68 negative edges among five MODs (Figure 4B), with a modularity of 0.41, suggesting a strongly associated community among the major communities which were mainly positive synergies. MOD0, MOD3 and MOD4 were strongly connected with other MODs with 100, 98 and 65 positive edges, respectively. There were more negative edges between MOD1 and MOD4, indicating a negatively competitive relationship among genera. Genera involved in carbon, nitrogen and sulfur metabolism were widely distributed in each module. *Flavobacterium*, *Dechloromonas*, *Massilia* in MOD0 participated in denitrification, nitrogen reduction and sulfate reduction (Tanikawa et al., 2018; Chang et al., 2019). The diversity of metabolic function makes it most closely related to other MODs. MOD3 were primarily denitrifying bacteria, such as *hgcI_clade* (Herrmann et al., 2015; Luo et al., 2018). Conversely, ecological modules with different functions often exhibit a tendency to compete for limiting nutrients (Tate, 2020). This competitive relationship may also exist between MOD1 and MOD4. MOD1 was dominated by *Limnohabitans*, *Planktothrix*, and other genera with both carbon and nitrogen metabolism function, whereas the MOD4 was ruled by nitrifying bacteria such as *Prosthecobacter*, *Nitrosomonas*, and *Nitrospira*. In addition, *Hydrogenophaga* and *Limnohabitans* in MOD1 and MOD4 participate in sulfur metabolism (Herrmann et al., 2015; Luo et al., 2018). Conjecturally, bacterial communities participated in nitrogen and sulfur cycles were potentially affected by the pollution of antibiotics and nitrogen in karst river.

### 3.3. Assembly mechanisms of bacterial community in karst river

The bacterial community assembly processes were consisted of deterministic processes (heterogeneous selection and homogeneous selection) and stochastic processes (dispersal limitation, homogenizing dispersal and drift) (Stegen et al., 2013). Deterministic processes represented the influence of environmental factors such as physical and chemical indicators on microbial community, while stochastic processes were related to spatial variation. In general, deterministic processes were dominant in small spatial scale while stochastic processes dominated in a larger spatial scale (Dini-Andreote et al., 2015). However, frequent anthropogenic impacts and intense variation in karst hydrodynamic conditions produced high variance and increased the influence of stochastic processes.

**Figure 5:**
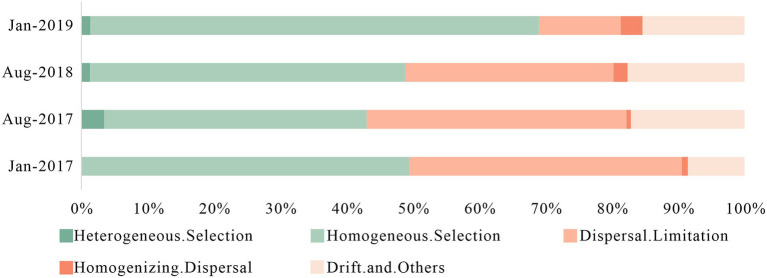
Assembly processes of bacteria community in karst river at four sampling times (Jan-2017, Aug-2017, Aug-2018, and Jan-2019) in karst river. Deterministic processes were composed of heterogeneous selection and homogeneous selection, and stochastic processes were composed of dispersal limitation, homogenizing dispersal, drift and other effects.

In Figure 5, stochastic processes had more advantage in Aug-2017 (56.96%) and Aug-2018 (50.66%), and primarily affected by dispersal limitation and drift effects. Stochastic processes played a major role in microbial community assembly with less environmental pressure (Dini-Andreote et al., 2015). Lower antibiotic concentrations in summer reduced the survival pressure for less resistant bacteria, which increased their relative abundance. The type of antibiotics in Aug-2017 and Aug-2018 were remarkable different, and their additive, synergistic and antagonistic effects led to greater differences in community structure (De Liguoro et al., 2018; Liang et al., 2022). The rapid surface-underground exchange and the drastic environmental changes in karst river resulted in short adaptation and rapid turnover in summer, which benefited distinguishable bacterial communities as well. Deterministic processes enhanced in winter, and homogeneous selection was predominant, with 49.51 and 68.14% in Jan-2017 and Jan-2019, respectively, which was caused by the survival pressure of high concentration of antibiotics and DIN. Therefore, we suggested that the environmental selection caused by the multiple pressure of antibiotics and DIN created a more stable bacterial community with antibiotic resistance in winter. Consequently, despite the two-year sampling interval between Jan-2017 and Jan-2019, the structure of bacterial community showed a highly similarity, while the stronger survival pressure from pollutions led to a decline in the relative abundance of specific genera. The proportion of dispersal limitation in the stochastic processes decreased gradually, while the proportion of homogenizing dispersal increased, indicating that the composition of microbial community tended to be more similar over time.

### 3.4. Effects of environmental factors on the functions of bacterial community in karst river

**Figure 6:**
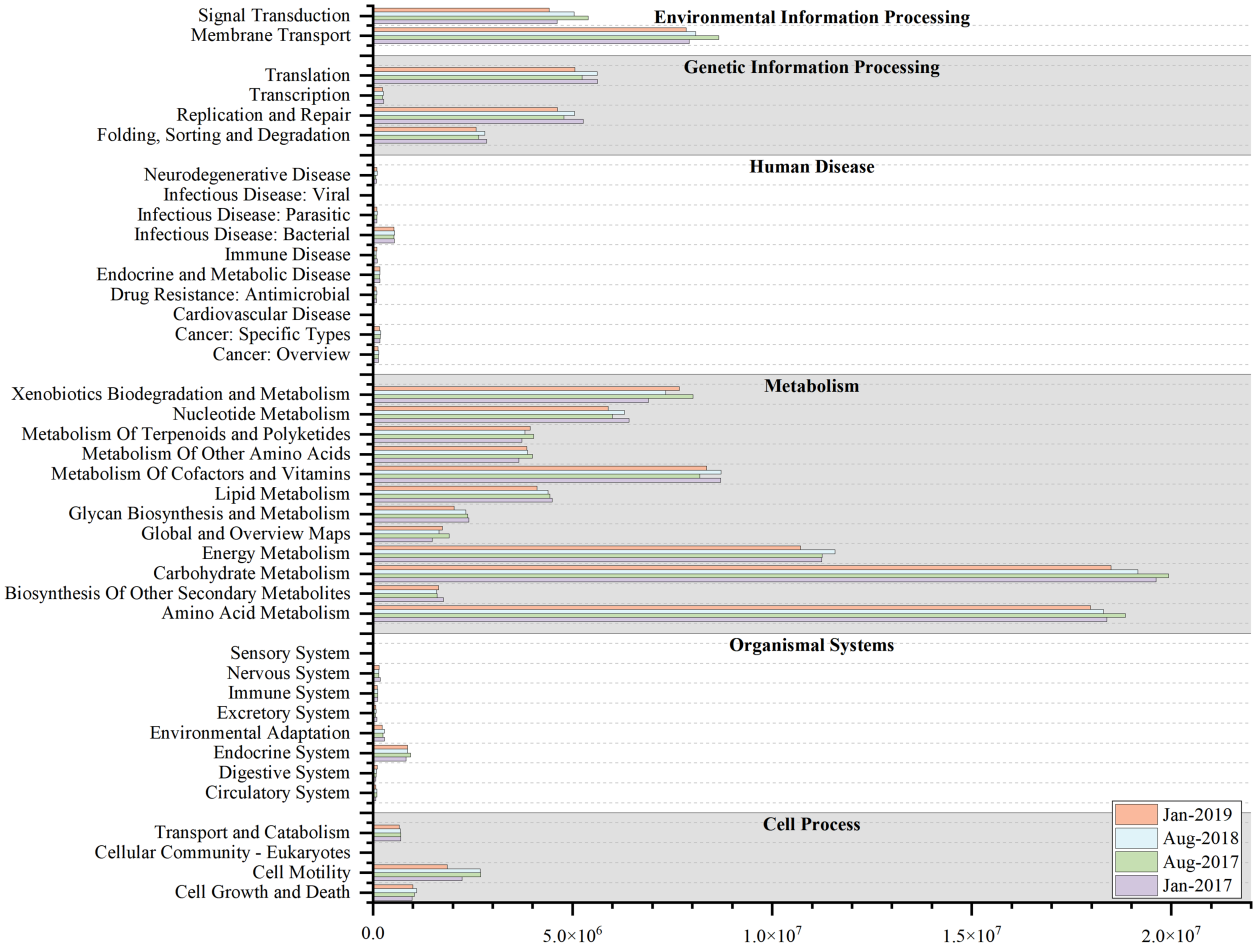
The Kyoto Encyclopedia of Genes and Genomes (KEGG) functional profiling at level-2 at four sampling time (Jan-2017, Aug-2017, Aug-2018 and Jan-2019).

Variations in microbial community were accompanied by alterations in community function (Zhong et al., 2018). The results of KEGG functional profiling revealed that the average relative abundance of 16 functional genes exceeded 1%, which were mainly sub-functions of metabolism, environmental information processing, and genetic information processing and so on (Figure 6). Among them, the average relative abundance of energy metabolism ranged from 5.58 to 5.79% and was higher in summer, possibly due to nutrient limitation (Cline and Zak, 2015; Jiang et al., 2022). In winter, the copious nutrients conditions made nutrients more accessible to microorganisms, and hence the corresponding metabolism gene was less prevalent. However, nutrient availability was restricted by dilution effects in summer, and microorganisms maintained their growth by increasing the metabolic function gene (Jiang et al., 2022).

**Figure 7:**
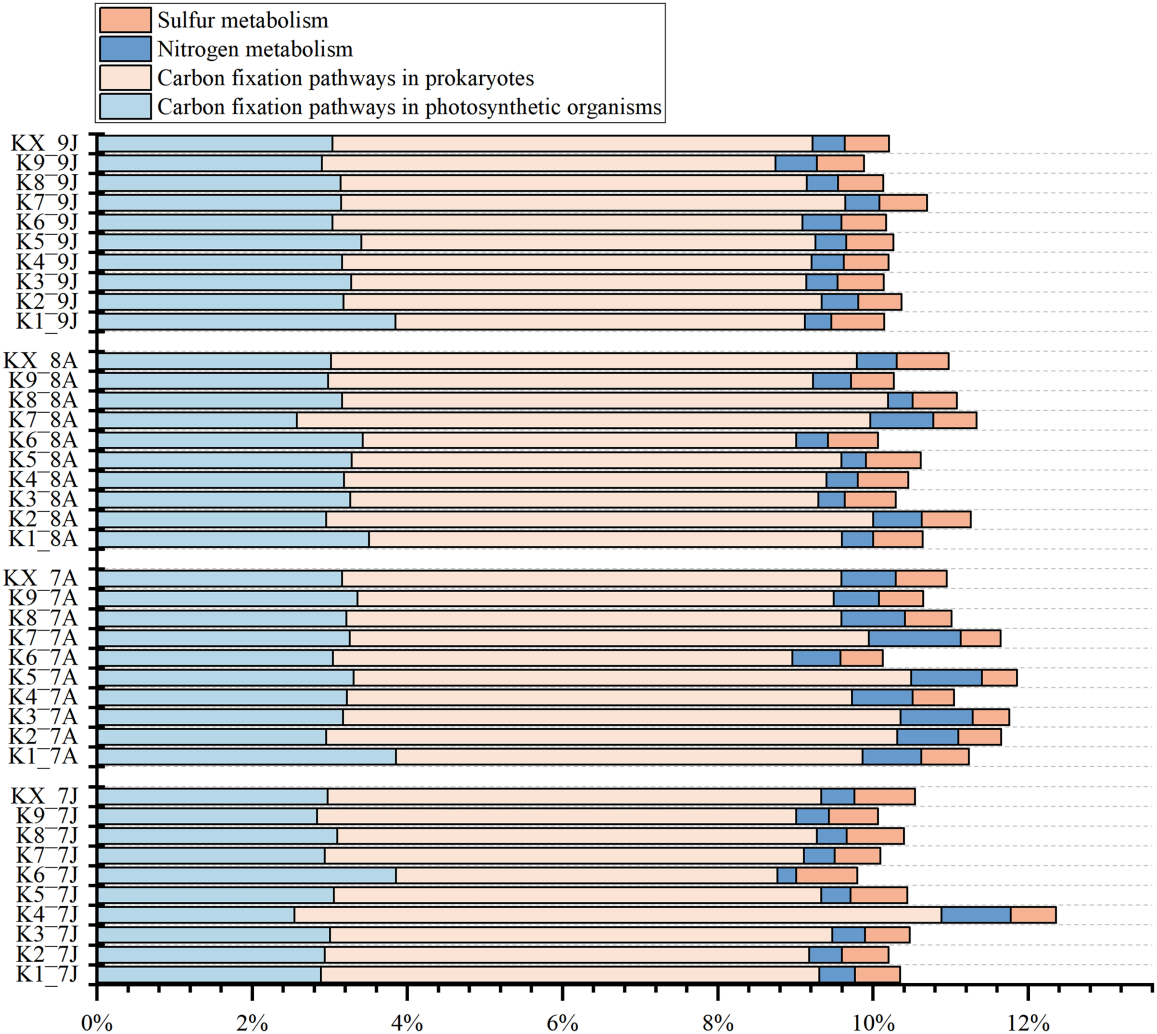
The stacked histogram of carbon, nitrogen, sulfur metabolic modules of Kyoto Encyclopedia of Genes and Genomes (KEGG) functional profiling at four sampling time (Jan-2017, Aug-2017, Aug-2018 and Jan-2019).

From the results of function profiling, 13 carbon fixation pathways, 5 nitrogen metabolism pathways, and 2 sulfur metabolism pathways were annotated (Figure 7; Supplementary Table 4). The carbon fixation pathways mainly included 7 carbon fixation pathways in prokaryotes (average relative abundance ranged from 5.98 to 6.58%) and 8 pathways in photosynthetic organisms (from 3.01 to 3.26%), such as reductive citrate cycle (M00173), dicarboxylate-hydroxybutyrate cycle (M00374), and reductive pentose phosphate cycle (Calvin cycle) (M00165), etc. The nitrogen metabolism pathways included nitrogen fixation (M00175), nitrification (M00528), denitrification (M00529), and 2 nitrate reduction to ammonium pathways (M00530 and M00531), with average relative abundances from 0.43 to 0.81%. The sulfur metabolism pathways included 2 sulfate reduction pathways (M00176 and M00596), with relative abundance ranging from 0.55 to 0.66%. But all the metabolism pathways above showed no clear seasonal variations.

**Figure 8:**
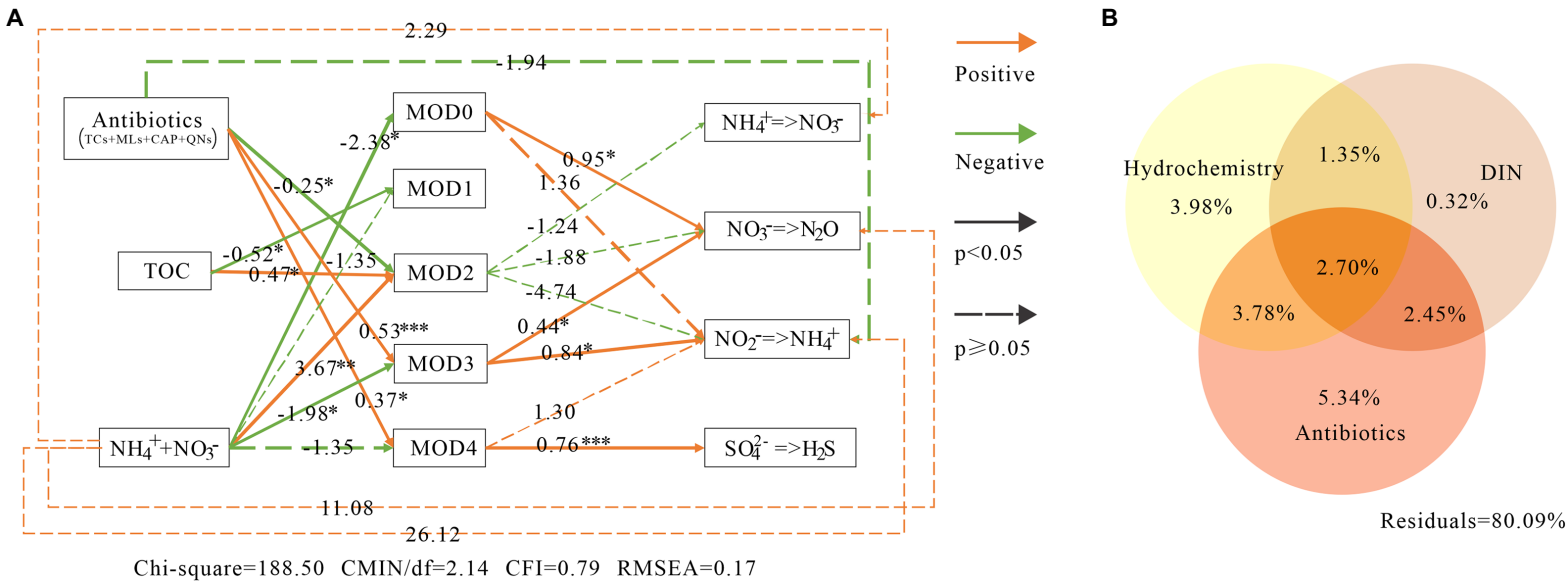
The environmental impacts on bacterial communities and their metabolic function in karst river. **(A)** Structure equation model (SEM) among environmental factors, modules and metabolic functions. Antibiotics were including tetracyclines, macrolides, chloramphenicol and quinolones. Green lines, orange lines indicated negative and positive impacts, respectively, and the associated numbers were the path coefficient value with p-values *p < 0.05, **p < 0.01, ***p < 0.001. **(B)** Variance partitioning analysis (VPA) diagram. The impact of antibiotics, DIN and hydrochemical factors on microbial communities. DIN included ammonium, nitrate and nitrite. Antibiotics included quinolones, lincomycin, tetracyclines, macrolides, sulfonamides and chloramphenicol. Hydrochemistry included temperature, pH, electronic conductivity, dissolved oxygen and total organic carbon.

SEM showed that MOD0, MOD3 and MOD4 contributed significantly to nitrogen and sulfur metabolism (Figure 8A). MOD0 was mainly composed of denitrification and nitrate reduction bacteria, so the denitrification was significantly influenced directly by MOD0, with path coefficient value of 0.95, and the nitrate reduction was not significantly influenced directly by MOD0. MOD3 also directly contributed significantly to nitrate reduction and denitrification, with coefficients of 0.84 and 0.44, respectively. Meanwhile, MOD4 played an important role in sulfate reduction.

On the other hand, the microbial community function was indirectly affected by environmental factors through certain ecological modules. Consistent with the result of VPA (Figure 8B), the antibiotics and DIN had significant impacts on microbial communities (with 5.34 and 0.53% explanation, respectively). MOD3 and MOD4 were resistant and promoted by the antibiotics. *Trichococcus* and norank_f__Leptotrichiaceae in MOD3 and *Acidovorax* and norank_p_Saccharibacteria in MOD4 carried resistance genes of tetracycline and multidrugs (Zhang et al., 2021), providing a stronger survival advantage in winter with higher concentration of antibiotics. DIN showed negative influence to MOD0, MOD3 and MOD4. The high nitrate content would produce significant inhibition or toxicity to bacteria (Cua and Stein, 2011), as well as to sulfate-reducing bacteria (He et al., 2010; Zhou and Xing, 2021). Besides, the high nitrate suppression to sulfate-reducing bacteria was diminished by the denitrifier (Zhou and Xing, 2021), which also explained the strong correlation between MOD4 and MOD0.

Denitrification, nitrate reduction and sulfur reduction were indirectly promoted by antibiotics *via* MOD3 and MOD4, whereas they were inhibited by DIN. As the substrate of nitrogen metabolism, DIN also indirectly promoted the process of nitrification, denitrification and nitrate reduction. SEM results showed that the total effect value of environmental factors on nitrification and nitrate reduction was −2.53 and 20.39, respectively. We suggested that the antibiotics and DIN inhibited nitrification, but strongly promoted the nitrate reduction, which lead a further accumulation of NH_4_^+^. Although the effects of antibiotics and inorganic nitrogen pollution on microbial ecological modules and their functions in karst rivers was indicated, the influence relationship of different ecological groups on environmental factors and the possible environmental utility was identified, the influence on metabolic process and the fluxes of nitrogen and sulfur still requires further researches such as long-term monitoring, isotope tests and other experimental methods.

## 4. Conclusion

The structure of microbial community with physicochemical properties altered considerably from winter to summer in karst ecosystem. Pollution such as antibiotics and inorganic nitrogen were introduced by anthropogenic activities from WWTP and agricultural discharges. Microbial communities in winter of 2 years interval were stable with higher similarity than those in summer of different year. Deterministic processes, homogeneous selection process specifically, occupied in winter due to the environmental pressure of high pollutants content, while stochastic processes had more advantages in summer with a significant extent of spatial heterogeneity due to intensive human activities and karst hydrodynamic condition. Pollution would indirectly affect the nitrogen and sulfur metabolism *via* microbial functional group. Furthermore, the environmental response and feedback of microbial communities’ element cycles needs more attentions and further measurable research.

## Data availability statement

The datasets presented in this study can be found in the NCBI repository (https://www.ncbi.nlm.nih.gov/), accession numbers PRJNA908735 and PRJNA936825.

## Author contributions

XG: contributed to the conceptualization, methodology, and writing. RH and BZ: participated in the formal analysis and discussion. CG: participated in the investigation. FL: participated in the topic and discussion of the whole study. All authors contributed to the article and approved the submitted version.

## Funding

This research was supported by the National Natural Science Foundation of China (Grant no. 42172336 awarded to XG), Guangxi key R & D program support (Grand no. Guike AB22080070 awarded to FL) and the Fundamental Research Funds for Central Universities (Grant no. 2652019077 awarded to XG).

## Conflict of interest

The authors declare that they have no known competing financial interests or personal relationships that could have appeared to influence the work reported in this paper.

## Publisher’s note

All claims expressed in this article are solely those of the authors and do not necessarily represent those of their affiliated organizations, or those of the publisher, the editors and the reviewers. Any product that may be evaluated in this article, or claim that may be made by its manufacturer, is not guaranteed or endorsed by the publisher.

## Footnotes

^1^ http://www.drive5.com/uparse/

^2^ http://www.mothur.org/wiki/Classify.seqs

^3^ https://github.com/picrust/picrust2/

^4^ https://github.com/taiyun/corrplot

^5^ https://callr.r-lib.org/

^6^ https://github.com/vegandevs/vegan

^7^ https://github.com/DaliangNing/iCAMP1

^8^ https://cran.rstudio.com/package=pheatmap

^9^ https://www.r-project.org

^10^ https://gephi.org/

^11^ http://nynct.guizhou.gov.cn/xwzx/zwdt/201712/t2017120525041433.html
