# Supplementary material for: Seasonal variations of microbial community structure, assembly processes, and influencing factors in karst river
Source: Front Microbiol. 2023 Mar 23;14:1133938. doi: 10.3389/fmicb.2023.1133938 (PMC10075313; doi:10.3389/fmicb.2023.1133938)
Supplement: Supplementary file 2 [file Data_Sheet_1.docx]

## Supplementary materials


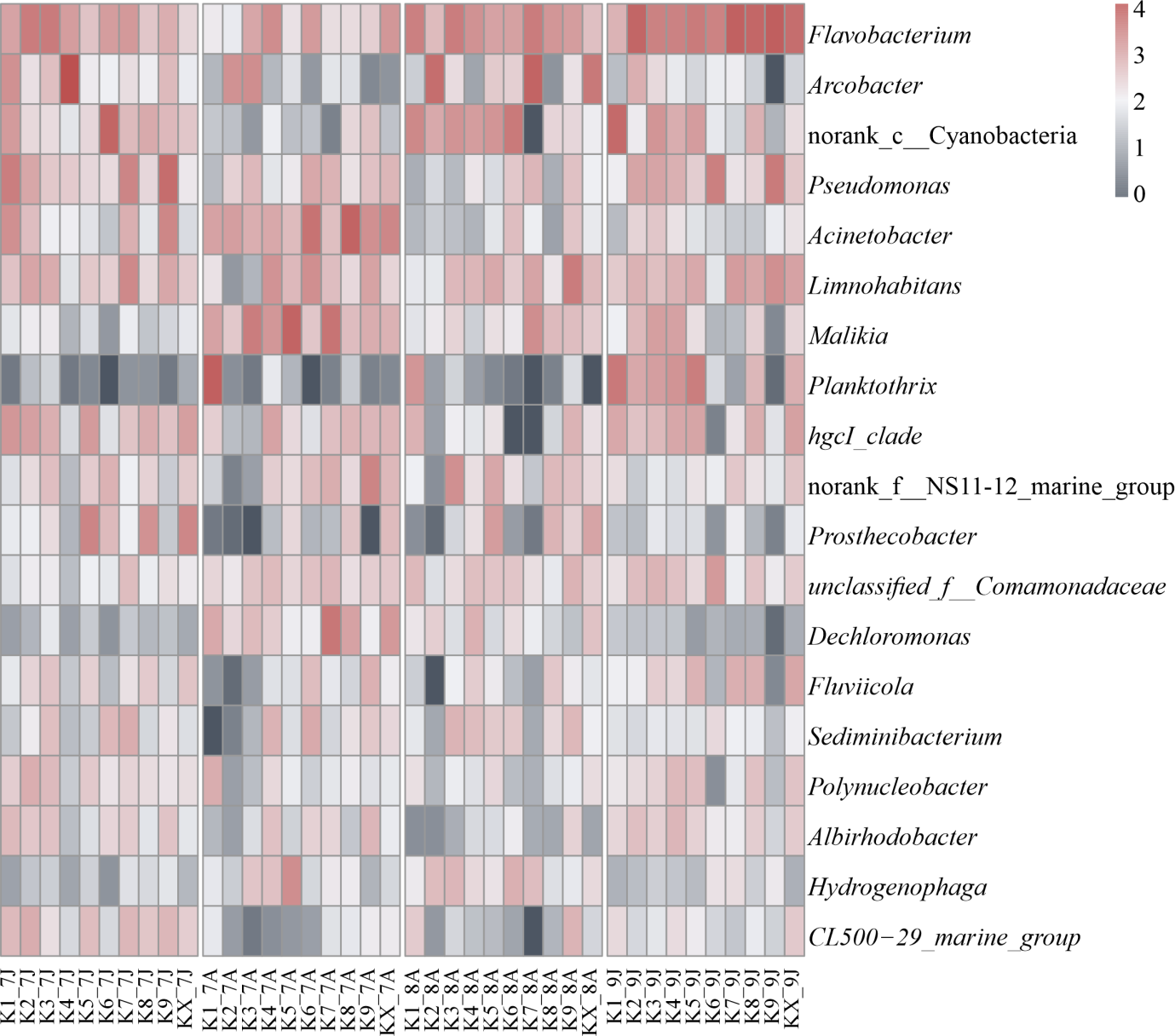


**Supplementary Figure 1**. The heatmap of dominant genus with relative abundance above 1% in karst river at four sampling times (Jan-2017, Aug-2017, Aug-2018 and Jan-2019). Genus abundance were normalized by lg(x+1).
